# Controversial aspects of imaging in child abuse: a second roundtable discussion from the ESPR child abuse taskforce

**DOI:** 10.1007/s00247-023-05618-5

**Published:** 2023-03-07

**Authors:** Michael Paddock, Arabinda K. Choudhary, Annmarie Jeanes, Kshitij Mankad, Inès Mannes, Maria Raissaki, Catherine Adamsbaum, Maria I. Argyropoulou, Rick R. van Rijn, Amaka C. Offiah

**Affiliations:** 1 Medical Imaging Department, Perth Children’s Hospital, Perth, WA Australia; 2 Division of Paediatrics, University of Western Australia, Perth, WA Australia; 3 Department of Oncology & Metabolism, University of Sheffield, Sheffield Children’s NHS Foundation Trust, Sheffield, UK; 4 Department of Diagnostic Radiology, University of Arkansas for Medical Sciences, Little Rock, AR USA; 5 Department of Paediatric Radiology, Leeds Children’s Hospital, Leeds Teaching Hospitals NHS Trust, Leeds, UK; 6 Department of Radiology, Great Ormond Street Hospital NHS Foundation Trust, London, UK; 7 Paediatric Radiology Department, AP-HP, Bicêtre Hospital, Le Kremlin-Bicêtre, Paris, France; 8 Radiology Department, Medical School, University Hospital of Heraklion, University of Crete, Crete, Greece; 9 Faculty of Medicine, Paris-Saclay University, Le Kremlin Bicêtre, Paris, France; 10 Department of Radiology, Medical School, University of Ioannina, Ioannina, Greece; 11 Department of Radiology and Nuclear Medicine, Emma Children’s Hospital, Amsterdam UMC, University of Amsterdam, Amsterdam, the Netherlands; 12 Department of Radiology, Sheffield Children’s NHS Foundation Trust, Sheffield, UK

**Keywords:** Child abuse, Children, Physical abuse, Infants, Radiography, Fractures, Head, Magnetic resonance imaging, Spine

## Abstract

This second roundtable discussion was convened at the 56th European Society of Paediatric Radiology (ESPR) 2022 Annual Meeting in Marseille, France, to discuss controversial aspects of imaging in child abuse. The following topics were discussed:

- Fracture dating—the published literature is broadly similar with respect to the identification of the radiographic stages of bony healing. The non-expert/general radiologist is encouraged to use broad descriptors of fracture healing (acute, healing or old) within their reports, rather than attempting to date fractures. The more experienced/expert radiologist, who may provide a timeframe/range to assist the courts, should be aware that any published timeframes are not absolute and that recent research indicates that the rate of healing may differ according to the bone affected and the age of the patient.
- Whole spine imaging in suspected abusive head trauma—this is recommended to enable a complete assessment of the neuraxis when abusive head trauma is suspected or diagnosed, particularly in the presence of intracranial and cervical subdural haemorrhage and cervical ligamentous injury.
- Cranial imaging in suspected physical abuse—both computed tomography (CT) and magnetic resonance imaging (MRI) remain complimentary depending on the clinical context in which they are used with CT remaining first-line in the assessment of children with (suspected abusive) head trauma prior to an early MRI. MRI is superior in its assessment of parenchymal injury and may be employed as first-line in age appropriate asymptomatic siblings of a child with suspected physical abuse.

## Introduction

The recognition of child abuse is challenging and complex and remains an emotionally charged field in which to work. As such, controversies continue to exist. According to the 2013 World Health Organisation European report on preventing child maltreatment, it has been estimated that child maltreatment contributes to the ‘premature death of 852 children under 15 years every year in the European Region’ with ‘18 million children suffering from sexual abuse, 44 million from physical abuse and 55 million from mental abuse’ out of an approximate 190 million children [1]. Radiologists play a pivotal role in the recognition of the radiological manifestations of physical abuse and may be the first healthcare professional to raise the suspicion of inflicted injury.

The first roundtable discussion by Boal et al. in 2001 [2] centred on the American child protection system; federal funding for research and development in child protection; and allocation of resources to keep children safe. The purpose of this second roundtable discussion was to gather international experts to share knowledge, understanding and experience and was convened at the 56th European Society of Paediatric Radiology (ESPR) 2022 Annual Meeting in Marseille, France. The aim of this publication is to share this expert-level discussion of challenging and controversial issues in the reporting of radiological imaging for suspected physical abuse for the benefit of the wider paediatric radiology community.

The following questions were posed:

- Benign enlargement of the subarachnoid spaces (BESS) and the presence of subdural haemorrhages: When to investigate for abuse?
- Should all siblings/household members less than 2 years old of a child with suspected physical abuse be routinely investigated?
- Is it sensible for radiologists (general and expert) to attempt to date fractures?
- Why, and in which scenarios should whole spine imaging be performed?
- Should head computed tomography (CT) be indicated for all infants and young children investigated for suspected abuse?

The detailed discussions regarding BESS and subdural haemorrhage, and the screening of siblings less than 2 years of age, will be published separately and readers are referred to these publications when they are available. The discussions regarding fracture dating, spinal imaging in abusive head trauma and routine cranial imaging in suspected physical abuse are presented herein.

## Discussion

### Is it sensible for radiologists (general and expert) to attempt to date fractures?

#### How common are fractures in physically abused children?

Radiologists play a vital role in the detection and assessment of physical abuse. Fractures are the second most common injury after bruising in children who have suffered physical abuse [3] and have been recorded in as many as 55% of these children, with 18% having sustained multiple fractures [4]. Fractures due to abuse may be clinically occult and are most common in infants under 18 months of age. Several studies have reported that 25 to 56% of all fractures in children aged less than 1 year were abusive [5]. The acquisition of a radiographic skeletal survey according to relevant international guidance (the Royal College of Radiologists (RCR) and Society and College of Radiographers (ScoR) [6] endorsed by Royal College of Paediatrics & Child Health (RCPCH) and the ESPR [7]; and the American College of Radiology (ACR) appropriateness criteria [8]) is advised in all children under the age of 2 years who are suspected of having been physically abused [9].

The commonest sites of abusive fractures are the long bone shafts [10], the ribs and the skull (the discussion of abusive skull fractures is beyond the scope of this article). Certain fractures/fracture patterns are known to be highly specific for abuse, e.g., the classic metaphyseal lesion (CML), rib fractures and the presence of multiple fractures at different stages of healing (and therefore ages) in the absence of an alternative explanation. Different patterns of injury are seen depending on the age of the patient: rib fractures and CMLs are more common in infancy with long bone shaft fractures more common in older children. By contrast, long bone shaft fractures are low-specificity injuries for abuse given that they are radiologically indistinguishable from those with an accidental aetiology.

No fracture/fracture pattern is pathognomonic of physical abuse. Determining whether a fracture is accepted to be accidental or more likely inflicted can often be challenging and is largely dependent upon the patient age, the developmental level of the child and the clinical history (i.e. the proffered mechanism of injury) provided by the parent/carer. Any fracture in a dependent, non-ambulant child is concerning for an inflicted (abusive) injury [11]. Moreover, the identification of any fracture in the context of suspected child abuse is significant and indicates the application of significant and inappropriate forces. Abuse may also be suggested when an injury is inconsistent with the accepted/usual mechanisms required to result in a specific fracture/fracture pattern based on the radiologists’ knowledge and experience of reporting (witnessed) accidental fractures in children where the mechanism is known.

#### Dating fractures: why?

In cases of suspected physical abuse, clinical histories can be inaccurate or absent and injuries are (often reported to be) unwitnessed. As such, it may be difficult to define when a fracture has occurred. Radiologists are often asked to date fractures by those involved in the care of the child (e.g. the paediatrician, the police, the Court) to confirm or refute the carers account of when the injury occurred. From a legal perspective, the timing of any injury may be of paramount importance which may influence decision-making regarding the potential pool of perpetrators and ultimately, the placement of the child into a place of safety.

#### Fracture healing: how?

From a histopathological perspective, long bone fractures heal in a predictable fashion with several distinct pathological phases of bone healing: haematoma formation; inflammatory response; callus formation and organisation; ossification; and remodelling. These phases of bone healing are manifest radiologically as soft tissue swelling, subperiosteal new bone formation (SPNBF), blurring/loss of fracture line, soft callus formation, hard callus formation and bony remodelling which can be used by radiologists to date fractures [12].

#### Dating fractures: what is the evidence?

Before discussing the pros and cons of whether radiologists should attempt to date fractures, the reasons behind some of the disparities between the published timetables need to be explored further.

In 1998, O’Connor and Cohen published a timetable of the radiological changes of fracture healing in the first edition of *Diagnostic Imaging of Child Abuse* by Paul Kleinman [13]. Since its publication, this timetable has been widely used by radiologists as a ‘guide’ to fracture dating and is still regularly referenced in the published literature. However, this work was based on the authors’ own observation and experience rather than peer-reviewed scientific evidence. In addition, the range of patient ages, the location of the fractures and whether the injuries were accidental or inflicted were not reported. The published literature on fracture dating is otherwise limited to a few small studies and texts, most of which include suggested timetables for the phases of fracture healing (Table 1) [13–23]. Whilst there are some similarities between some of the suggested timetables, there are also significant differences which, from a practical and legal perspective, question their accuracy and validity in assisting fracture dating. Moreover, it is important to note that these studies are retrospective and that the imaging used to estimate healing was not designed to determine the ‘first sign’ of each healing stage, but rather was used to follow the stages of healing from a clinical perspective, i.e. by the treating orthopaedic surgeon to facilitate clinical decision-making/management. As such, this may explain some of the apparent inconsistencies between the published timetables.

**Table 1:** Summary of the published studies and texts assessing fracture healing on radiographs in patients up to 17 years of age

Author(s)	Cumming [14]	Yeo and Reed [15]	O’Conner and Cohen (in Kleinman) [13]			Islam et al. [16]	Offiah and Hall [17]	Halliday et al. [18]	Prosser et al. [19]			Walters et al. [20]	Warner et al. [21]	Fadell et al. [22]	Crompton et al. [23]
Year	1979	1994	1998			2000	2009	2011	2012			2014	2017	2017	2021
Age	Newborn	1 to 14 years, infants excluded; 10 patients < 4 years of age)	Unknown			1 to 17 years, infants excluded, mean age 8 years	< 4 years	14 days to 44 months, median 5 months	0 to 6 years, mean 4.8 years			0 to 3 months	< 12 months	0 to 6 months	< 33 months
Bone or injury type	Birth-related fractures											Clavicle		Clavicle	Femur
Number of patients	23 infants											131 infants		61 infants	30 children
			Early	Peak	Late				Early	Peak	Late				
Radiological feature															
Soft tissue swelling							< 7 to 10 days		1 day	1 to 2 days	31 days				
Resolution of soft tissue swelling			2 to 5 days	4 to 10 days	10 to 21 days			10 days							
Fracture gap widening (range)						*28 to 42 days, 56% (14 to 8 days)	> 7 days								
SPNBF (range)	9 to 10 days (7 to 11 days)	Mean 11 days (7 to 21 days)	4 to 10 days	10 to 14 days	14 to 21 days	*28 to 49 days, 100%; earliest 14 days	7 to 10 days	11 days, > 90%; earliest 4 days	5 days	15 to 35 days	96 days	> 10 days; earliest 7 days; 8 days, 50%	> 9 days (7 to 130 days)	11 days (7 to 49 days)	> 12 days, 83.3%; 7 to 11 days, 22%
First callus			10 to 14 days	14 to 21 days		*28 to 49 days, 100%	*7 to 42 days		12 days	22 to 35 days	66 days	> 15 days; 13 days, 50%; earliest 9 days; mean 18 days	9 to 14 days (9 to 130 days)	11 days (11 to 61 days)	> 27 days, 89.5%; 15 to 26 days, 50%
(Hard) callus density > cortex			14 to 21 days	21 to 42 days	42 to 90 days	*91 days, 90%		20 to 106 days	19 days	≥ 22 days	96 days	Mean 42 days			
Bridging (range)		*18.2 days (10.5 to 25.9 days)				*91 days, 50%			19 days	*35 days (≥ 36 days)	300 days		15 to 51 days	22 days (20 to 63 days)	
Remodelling (range)		*56 days	*91 days	*365 days	* > 730 days	*63 days, 50%			*42 to 49 days	*35 days (≥ 36 days)	421 days		> 51 days	49 days (35 to 151 days)	26 to 37 days, 26.7%; > 42 days, 100%

*SPNBF*, subperiosteal new bone formation

^*^Converted to days from weeks, months or years to facilitate comparison

The authorship and the ESPR Child Abuse Taskforce do not endorse any specific timeframe cited in this table. The relevant information has been presented to provide the readership with a comprehensive overview of the relevant published studies and texts

It is accepted that bone healing is faster in infants and young children when compared to adults. Additionally, it is well recognised that in the context of physical abuse, fractures are more commonly seen in children under the age of 3 years with a least half occurring in children less 12 months of age [24, 25]. Yet, several published timetables for fracture healing included older children and adolescents in their analyses [15, 16, 19]. There are 7 studies which concentrated on fracture healing in children aged less than 5 years [14, 18, 20–23, 26]. Of these, only 4 assessed fracture healing in children under the age of 3 years of age [20–23].

Yeo et al. assessed fracture healing in femoral fractures in children from birth to 14 years of age, of which only ten children were under 4 years of age [15]. Islam et al. assessed the morphology and rate of healing of forearm fractures in children up to 17 years of age (mean age: boys, 8.4 years; girls, 7.1 years) [16]. Both studies excluded children under the age 12 months from their analyses—the group most at risk for abusive fractures. Prosser et al*.* analysed accidental long bone fractures in 63 children under 72 months (6 years) of age. Children under 12 months were included but the mean age was 4.8 years suggesting that most of the children were older than those typically seen with abusive fractures [19]. It is noted that the peak period for SPNBF varied significantly between these three studies: 1.6 weeks (11.4 days) [15]; 4 to 7 weeks (28 to 35 days) [16]; and 15 to 35 days [19], respectively. However, the earliest radiographically detected SPNBF was similar for both Prosser et al. [19] and Yeo et al. [15] at 5 days and 7 days, respectively.

In addition to age, there are a number of other important variables which may influence the rate and radiological detection of fracture healing. It is accepted that limb immobilisation promotes fracture healing in adults by stabilising the bone and surrounding musculature. However, the effect of limb immobilisation on fracture healing in children has not been confirmed. In those studies which assessed long bone fractures, the authors consistently reported that the presence of a cast and fracture immobilisation potentially influenced the detection of the radiographic features of fracture healing [15, 18, 19, 23].

Fracture healing is also influenced by the type of fracture, i.e. complete, incomplete or displaced: only a single study differentiated the pattern and rate of fracture healing according to fracture type [21].

In an attempt to address some of these factors (patient age, cast, immobilisation, fracture type), Walters et al. [20] and Fadell et al. [22] evaluated the pattern and rate of fracture healing in infants with birth-related clavicular fractures. Both studies assessed four phases of bone healing: SPNBF, callus, bone bridging and remodelling. Their results confirmed a predictable pattern of healing for clavicular fractures and published suggested timetables for fracture healing with good concordance between the two studies for SPNBF (Table 1). Fadell et al. suggested that their timetable for clavicular fracture healing could be used to assist dating of other long bone fractures in cases of SPA [22], whereas Walters et al. questioned whether their proposed timetable was applicable to all long bones [20].

It is well recognised that upper limb fractures heal faster than lower limb fractures in adults [26–28]. There is evidence that rates of fracture healing also differ according to fracture site in children. In 2011, Malone et al. compared the healing rates of tibial and radial fractures in 107 infants and young children [26] and concluded that patient age and fracture location influenced the rate of fracture healing with forearm fractures healing faster than tibial fractures. In 2021, Crompton et al. assessed femoral fractures in children less than 4 years of age with the aim of evaluating differences in healing rates between femoral and birth-related clavicular fractures [23]. They reported that the pattern of healing was similar but that the SPNBF and callus stages of femoral fracture healing in children up to 3 years appeared to lag behind those published for clavicular fractures. Both of these studies indicated that the rate of fracture healing in children is influenced by fracture site and cautioned the use of published timetables based on the rate of healing of long bone fractures at other fracture sites.

The 4 more recent published studies [20–23] support some of the findings from the earlier published literature: a predictable pattern of radiological fracture healing for both long bone and clavicular fractures; good concordance regarding the first appearance of SPNBF, this usually being present by 11 days (range 7 to 12 days); and good concordance for bony remodelling, this being present by 42 days (range 26 to 51 days) in all studies where it was assessed. There was no consistency between studies when considering callus formation (first or mature) or the bridging phases of bone healing.

#### Is it sensible for radiologists to attempt to date fractures?

Most radiologists (whether specialist paediatric or general) would usually feel confident to provide an opinion as to whether a fracture is acute, healing or old based on their clinical experience of reporting accidental fractures in children where the time of injury is reported/known. However, in the context of suspected physical abuse, treating clinicians, the police and/or the courts apply pressure to radiologists to provide a timescale, or to ‘date’ a fracture(s), in an attempt to gain additional evidence to support the decision to commence formal legal investigations.

Most radiologists involved in imaging children will be aware of the timetable on fracture dating published by O’Connor and Cohen in 1998 [13]. However, it is unlikely that the ‘general paediatric radiologist’ will be aware of the broader published literature and its strengths and weaknesses unless they have a specific interest or expertise in reporting imaging performed for suspected physical abuse. Reporting radiologists who provide specific timescales for fractures run the risk of being asked to attend court to justify one’s opinion, especially where there is discrepancy with other experts. Given the lack of paediatric radiologists, both in general and specifically those willing to provide expert opinion for the Courts in cases relating to child protection [29], in the UK at least (noting that there are differences in legal practices across the European Union), one also runs the risk of being treated as an expert by the Court rather than as a witness of fact (professional witness). Ultimately, experts are expected to command a broad knowledge of the literature.

Consequently, although the (general/non-expert paediatric) radiologist may be involved in cases of suspected physical abuse in their own practice (e.g. raising the possibility of physical abuse at first presentation), providing specific timescales for fractures is not usually necessary and, where possible, should be avoided. As such, general/non-expert radiologists are advised to provide broad timescales—i.e. acute, healing or old—and defer to the experience of expert/more experienced specialist paediatric radiologists in this regard.

#### Should ‘experts’ attempt to date fractures?

An expert witness is appointed to assist the Court in providing advice on matters outside of its knowledge and can be defined as a person whose level of specialised knowledge or skill in a particular field qualifies them to present their opinion about the facts of a case.

In the context of suspected physical abuse, the proceedings aim to determine the facts and to assist the Court in its decision-making with respect to the safety and welfare of the child. Regarding timing or ‘dating’ of a fracture in these cases, experts appointed by the court may be requested to provide the most likely timeframe for any fracture identified. This opinion is expected to be based upon scientific evidence and not solely on individual clinical experience or knowledge. However, in practice, experts provide their opinion from reporting accidental fractures in children whereby the timing of the injury is known in addition to a broad knowledge of the published literature. Most experts have a wealth of personal experience in reporting radiographs in children with the typical radiologist reporting between 2500 to 6000 radiographs per annum amounting to approximately 50,000 to 100,000 radiographs over a 20-year career.

Clinical experience, although helpful, is not accepted by the courts as sufficient: any opinion must be supported by robust published evidence. Yet, as outlined above, the literature on fracture dating is limited, with only 7 studies analysing the rate of fracture healing in the age of children most at risk of physical abuse with more recent studies suggesting that the rate of fracture healing may vary by fracture site [23, 26].

There is frequently, and quite rightly, close scrutiny of expert evidence with regard to any timeframes provided. This can result in challenging and seemingly aggressive cross-examination, which is often regarded as unjustified, unfair or inappropriate by experts, and has been highlighted as one of the key factors for experienced paediatric radiologists being reluctant to undertake expert work [29]. The alternative is that experts do not attempt to provide timescales for fractures but simply opine as to whether a fracture is acute or healing. Given that the timing of fractures is often critical to the case, refusing to provide a timescale purely for fear of being challenged could be hugely detrimental to the court process and ultimately, the welfare of the child.

With respect to SPNBF and bone remodelling, there are broad similarities between more recent [20–23] and older published studies and texts [14, 17, 18], summarised in Table 1. Otherwise, it is suggested that timeframes for fracture dating should be kept broad with the caveat that any timeframes are not absolute and may differ according to bone affected. Further research with large collaborative multicentre studies is required in conjunction with clearly defined radiological consensus criteria with the aim of producing agreed timeframes for fracture dating in children.

### Why, and in which scenarios, should whole spine imaging be performed?

Reviewing the previous roundtable paper from 2001 [2], it would appear that our understanding of the mechanism of injury in abusive head trauma has not changed significantly. The need for, and extent of, spinal imaging and the significance of the imaging findings in the context of abusive head trauma have been recently emphasised [30, 31] with existing controversies summarised by Canty et al. [32] and published subsequent to the ESPR 2022 roundtable discussion.

Spinal vertebral fracture and cord injury of abusive aetiology are self-explanatory but rare. Much of the past literature based on radiographic and CT imaging rightfully considered spinal injury to be rare in abusive head trauma [33]. However, doubt has been cast on the aetiology of abusive head trauma considering that (even severe) brain injury from forceful shaking is possible without significant concomitant injury to the neck [34].

The need for, and the extent of, spinal imaging in abusive head trauma is further complicated by the variable availability of magnetic resonance imaging (MRI) and other related resources, such as sedation services, which are not uniform across centres which image children (including specialist paediatric institutions and general hospitals which also image adults) [32]. We discuss justifying full spinal imaging and the implications of potential spinal findings in children investigated for suspected abusive head trauma.

**Fig. 1:**
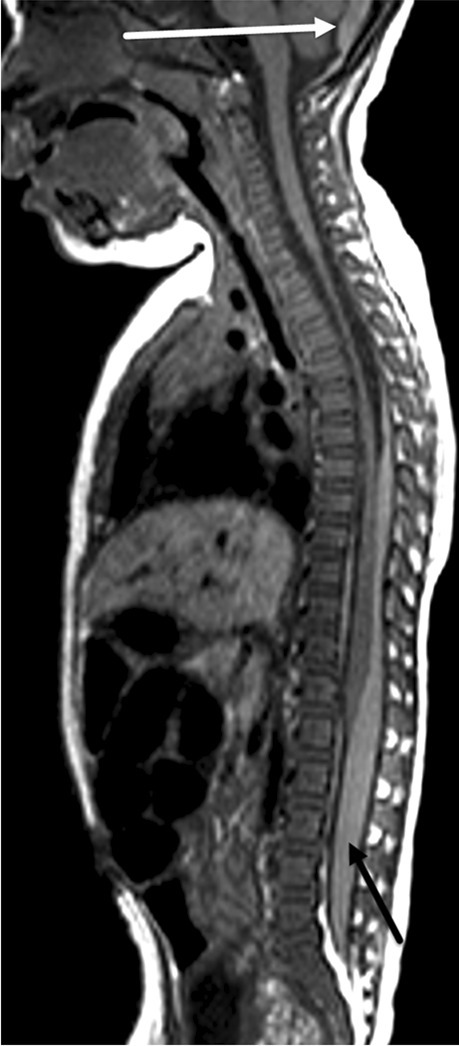
Selected sagittal T1 imaging of the whole spine in an 8-month-old boy with abusive head trauma demonstrates T1 hyperintense subdural haemorrhage within the posterior fossa (*white arrow*) and the thoracolumbar spinal canal (*black arrow*). There is no vertebral fracture or other evidence of local spinal or soft tissue injury

**Fig. 2:**
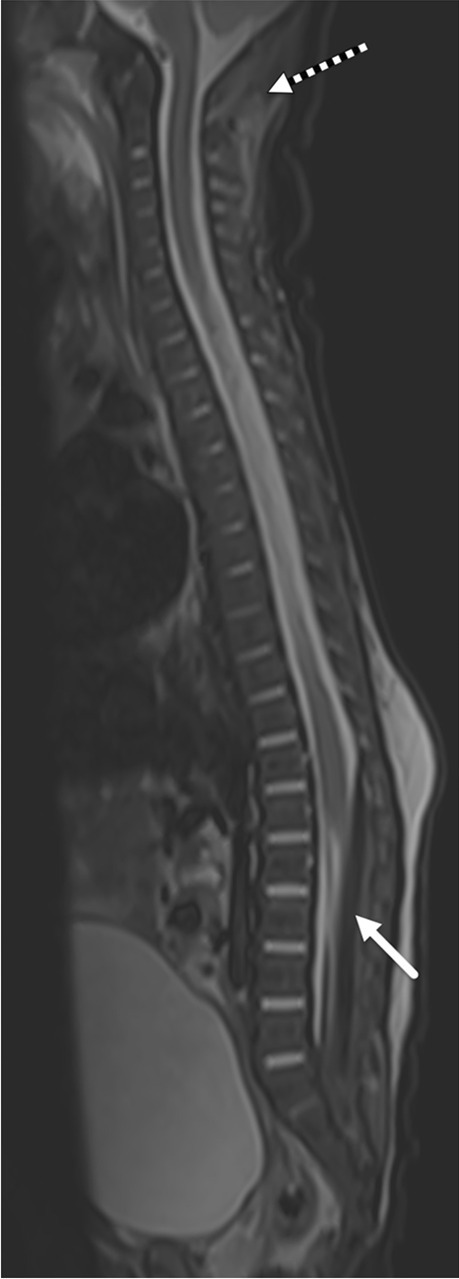
Selected sagittal short tau inversion recovery (STIR) imaging of the whole spine in a 3-month-old boy demonstrates T2 hypointense subdural haemorrhage in the lumbar spinal canal (*solid arrow*). The spinal canal subdural haemorrhage is not identified in the upper spinal canal and would have been missed had the lower spine not been imaged. There is also evidence of suboccipital spinal ligamentous injury (*dashed* *arrow*)

In abusive head trauma, the two primary imaging findings commonly identified on spinal imaging are subdural haemorrhage and spinal ligamentous injury [30, 35–37] (Figs. 1 and 2). Vertebral and cord injuries are rare. Spinal subdural haemorrhage is more commonly seen in the lower thoracolumbar region and may be missed if only the cervical and upper thoracic spines are imaged [35].

In a scenario whereby there was a positive finding on spinal MRI, such as spinal subdural haemorrhage or spinal ligamentous injury, but the required spinal region had not been imaged and such findings were not documented, there would be several questions to consider:

1. Did we miss a clinically significant finding which requires active clinical management?
2. Did we miss a finding that may help establish or refute the diagnosis of abusive head trauma?
3. What is the overall ‘value’ of this missed finding (where ‘value’ can be considered in terms of its clinical, forensic and medicolegal significance)?

#### Did we miss a clinically significant finding which requires active clinical management?

Apart from a case report in an adult [38], no significant adverse clinical outcomes of spinal subdural haematomas have been reported. The management of ligamentous injuries of the cervical spine varies across different institutions [31, 39]. In most centres, cervical spine immobilisation and stabilisation by way of collar are utilised if ligamentous injury is detected. Another challenge is to distinguish the clinical impact of these spinal injuries and their potential to mask the overall significant injury to the brain. In most of these cases, we can say that the evidence is still evolving and the clinical impact is probably not certain at this point in time.

#### Did we miss a finding that may help establish or refute a diagnosis of abusive head trauma?

We require a larger pool of evidence to draw statistically significant associations between injurious spinal findings and abusive head trauma to be able to confidently exclude the circularity argument. The accumulation and review of current evidence are encouraging [32]. In the published literature, there is a high percentage of spinal subdural haemorrhage in cases of abusive head trauma which is also statistically associated with significant injury to the brain, such as hypoxic ischaemic injury (HII) [36]. As the utilisation of spinal imaging in abusive head trauma increases, we are able to validate spinal injury findings across multiple institutions around the world which, in turn, may also help to validate a common mechanism of injury. Imaging may also help to refute other diagnoses—if diagnoses such as vitamin K deficiency, rickets, protein C/S deficiency, Ehlers-Danlos syndrome, and BESS are to be entertained, they will have to explain either spinal ligamentous injury or spinal subdural haemorrhage, either in isolation or combination.

#### What is the overall ‘value’ of this missed finding?

The ‘value’ of an imaging finding is derived from its ability to aid/make a diagnosis, refute alternate possibilities, establish further clinical management and, in cases of mortality, help to provide an assessment of risk to other at-risk/vulnerable family members. This finding may also aid any forensic investigation. The basic principle of what is ‘reasonable clinical practice’ at an institution for any other clinical diagnosis, based on available resources or policies, should also apply to the diagnosis of abusive head trauma.

An awareness of which findings may go undetected due to lack of available imaging will assist institutions in tailoring their diagnostic approach to abusive head trauma diagnosis alongside recent technological advances: whole body coils which enable the entire spine to be imaged simultaneously; and the development of newer sequences with reduced time parameters needed to complete spinal imaging in children.

#### Considering the above scenario, what findings will be missed if the spine is not imaged in the context of abusive head trauma? In which scenarios, should whole spine imaging be performed?

Where abusive head trauma is being considered, imaging of the whole spine would aid the diagnosis (of abuse), particularly in the presence of intracranial subdural haemorrhage. One of the authors (AKC) has never seen spinal subdural haemorrhage present without posterior fossa haemorrhage also being present; however, this combination has been reported in the literature [40].

If spinal imaging in the context of abusive head trauma is limited to the cervical spine, the presence of cervical ligamentous injury should prompt imaging of the whole spine given that is significantly associated with abusive head trauma [36]. Evidence of any additional spinal injury, such as bony or cord injury, should also prompt evaluation of the whole spine. If, on evaluation of cervical spine, spinal subdural haemorrhage is already identified in the cervicothoracic spinal canal, extension of imaging to include the remainder of the spine may not add anything further given that in most of the cases of spinal subdural haemorrhage, no other spinal injury is typically identified [35, 36]. However, this presumes that the patient does not have any clinical sign attributable to spinal cord compression or injury which would also be an indication to image the remainder of the spine.

In summary, imaging of the whole spine is recommended to enable a comprehensive evaluation of the neuraxis in the context of abusive head trauma [32]. Further studies are needed to understand the frequency and importance of these findings and their relationship to both abusive head trauma and accidental trauma.

### Should head CT be indicated for all infants and young children investigated for suspected abuse?

Despite the discrepancies between European countries, most agree on the necessity of a radiographic skeletal survey and cranial imaging in order to investigate children with suspected abuse [41]. When performed for this indication, it is worthwhile revisiting the advantages of CT and discussing its use in relation to suspected abusive head trauma: could we choose to perform only CT or MRI? What is the justification to continue exposing this cohort of children to ionising radiation when a non-ionising alternative may be available?

**Fig. 3:**
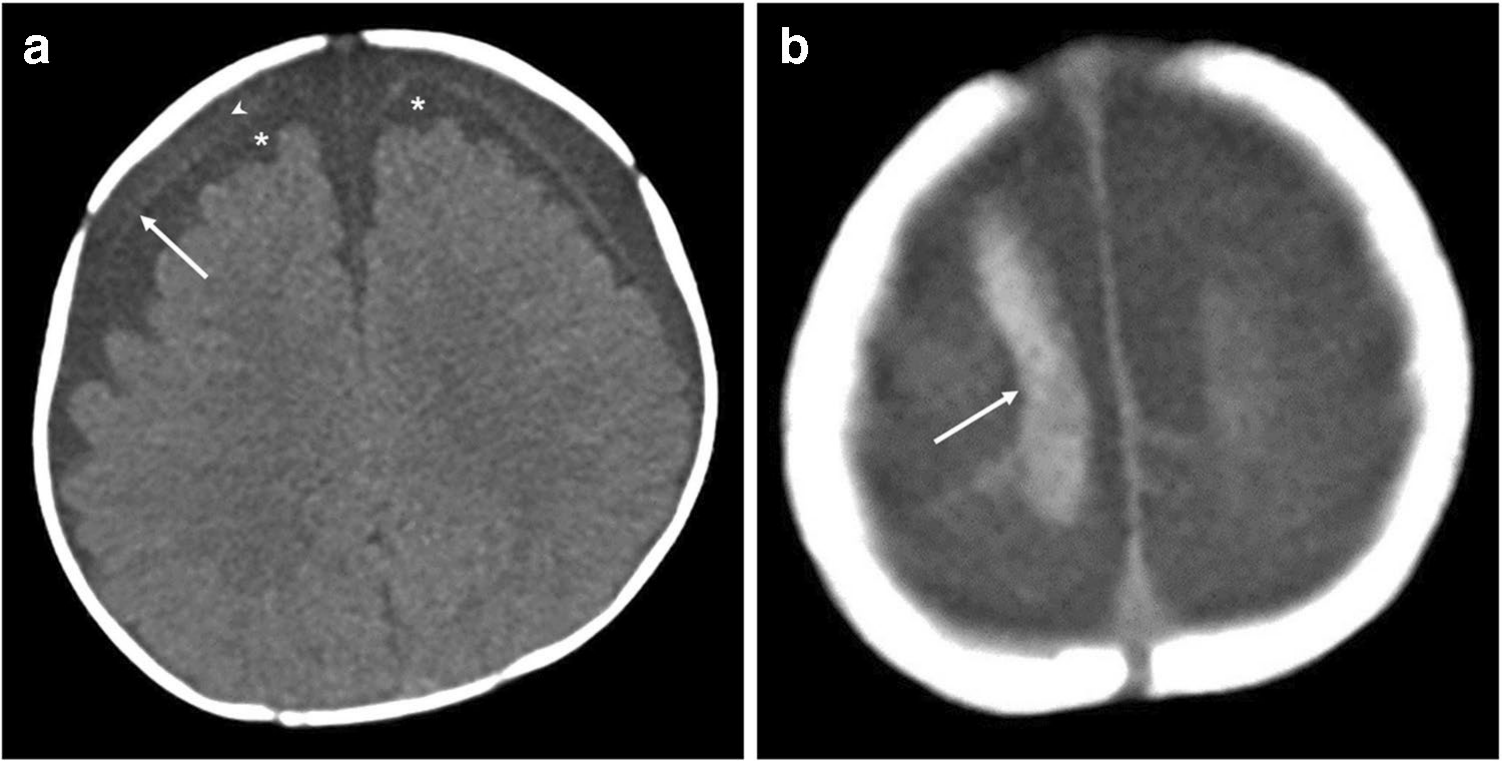
Selected axial head CT imaging (soft tissue window) in a 2-month-old boy with abusive head trauma demonstrates (**a**) bilateral benign enlargement of the subarachnoid spaces (*asterisks*) and bilateral subdural haemorrhages of mixed attenuation with slightly higher attenuation on the right (*arrowhead*). The arachnoid membrane separates the subdural collection from the enlarged subarachnoid spaces (*arrow*). **b** A rupture/thrombosis of a bridging vein at the right vertex (*arrow*)

The key neuroimaging findings which raise the suspicion for abusive head trauma are (1) haemorrhage, particularly multifocal subdural hematomas (convexity and interhemispheric fissure, Fig. 3) and occasionally subarachnoid and/or intraventricular haemorrhage; (2) rupture/thrombosis of bridging veins; (3) skull fractures, with or without scalp swelling; and (4) parenchymal injury, such as cerebral contusion, hypoxic ischemic injury and/or lacerations [42].

#### What are the advantages and disadvantages of CT?

**Fig. 4:**
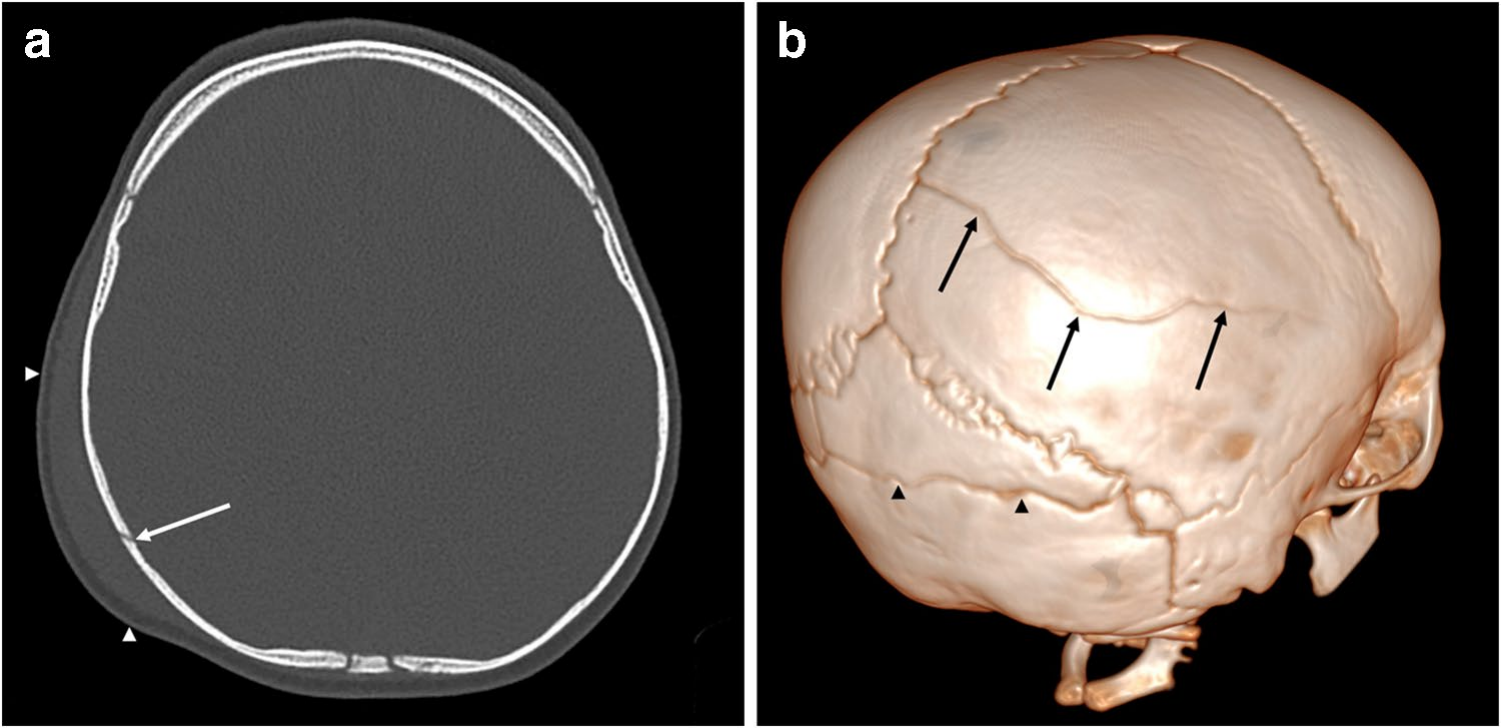
Head CT imaging in a 9-month-old male. **a** The selected axial slice (bone window) demonstrates a right parietal skull fracture (*arrow*) with overlying soft tissue swelling (*arrowheads*). **b** The 3D volume-rendered reconstruction (right posterior oblique view) from (**a**) also demonstrates the right parietal skull fracture (*arrows*). Note the accessory suture in the occipital bone (*arrowheads*) which is a normal anatomical variant alongside several intrasutural bones in both lambdoid sutures. This accessory suture can be differentiated from a fracture due to its ‘zigzag’ morphology, sclerotic margins (on multiplanar imaging) and regular interdigitations without overlying soft tissue swelling. Moreover, accessory sutures are commonly located in the occipital bone

The advantages include the following:

- It is available almost immediately in every centre (whether dedicated paediatric or not) and can be performed quickly and easily without sedation. A strict CT protocol must be followed: thin-section submillimetre slices without contrast from skull to vertex with three-dimensional (3D) volume-rendered reconstructions.
- It is efficient in the evaluation of subdural hematoma. Acute intracranial haemorrhage, which appears hyperdense/hyperattenuating, is easy to detect even for the lesser experienced radiologist (or clinician). Bridging vein rupture/thrombosis can also be identified on head CT as hyperdense clot at the vertex (Fig. 3).
- The identification of soft tissue swelling and the use of 3D reconstructions better help to distinguish between true fractures and normal variants (such as accessory sutures) when compared to skull radiographs [43] (Fig. 4), which is particularly relevant when considering that the probability of abuse in children with skull fractures is 30% [44]. Moreover, the diagnostic accuracy of CT with 3D reconstructions far exceeds that of radiographs in the diagnosis of skull fracture [45]. As such, skull radiographs could be excluded from the radiographic skeletal survey performed for suspected physical abuse in children less than 12 months of age given that they provide no further diagnostic information when CT with 3D reconstructions has, or is going to be, performed [45–47].

Nevertheless, head CT has some limitations:

- It exposes young children to ionising radiation; although with the advent of newer technologies, doses are less than they used to be alongside the strict application of the widely known As Low As Reasonably Achievable (ALARA) principle [48].
- Evaluation of the brain parenchyma is limited, particularly in the acute phase of abusive head trauma during which it can appear normal despite the presence of severe hypoxic ischemic injury—in this regard, MRI with diffusion-weighted sequences is far superior.

#### What are the advantages and disadvantages of MRI?

**Fig. 5:**
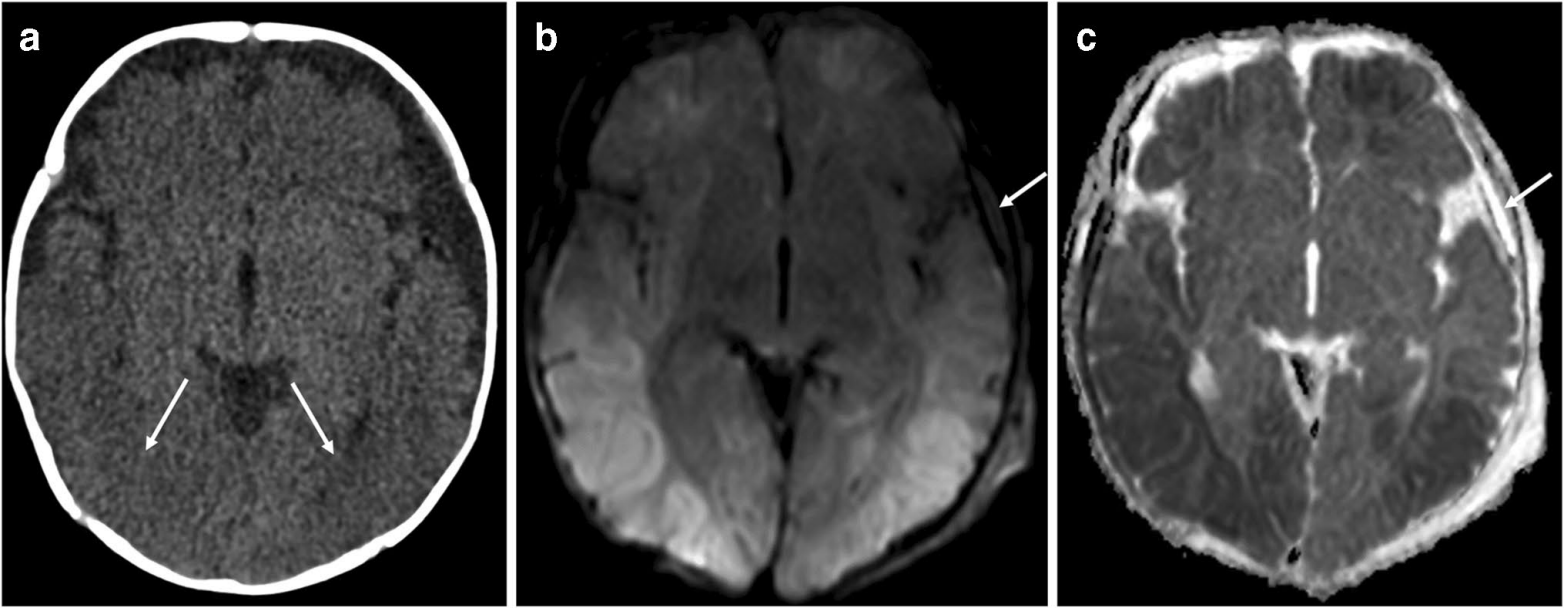
Selected cranial imaging in a 5-month-old girl. **a** The axial head CT (soft tissue window) demonstrates subtle predominately posterior (*arrows*) loss of grey-white matter differentiation. There is enlargement of the left extra-axial space without visible subdural haemorrhage. The axial MRI (**b**) diffusion-weighted imaging (DWI) and the (**c**) apparent diffusion coefficient (ADC) map, performed 12 h after (**a**), demonstrate corresponding recent diffuse hypoxic ischaemic injury in the same areas of grey-white matter differentiation loss visualised in (**a**) which are better depicted on MRI. An intermediate signal intensity small subdural collection (*arrow*) is visible in (**b**) with corresponding high signal intensity (*arrow*) in (**c**)

The advantages include the following:

- The diagnosis of parenchymal lesions, such as cerebral contusion, hypoxic ischemic injury, diffuse axonal injury and parenchymal lacerations—there is an indisputable superiority of MRI in this regard [49]. Early imaging with diffusion-weighted sequences typically reveals hypoxic ischemic injury that is not well demonstrated on CT (Fig. 5).
- The detection of bridging vein rupture/thrombosis can be detected on susceptibility-weighted imaging (SWI)/gradient echo imaging T2*-weighted imaging (GRE T2*). Normal bridging veins can be seen on standard T2-weighted imaging as low signal, regular, linear structures at the vertex. However, they lose their normal morphology when they rupture and appear irregularly thickened. The thrombosis of these ruptured veins can also be easily identified as low signal intensity along the pathway of the bridging vein, also known as the ‘tadpole’ or ‘lollipop’ signs which have been reported to have high diagnostic value [50].
- The analysis of the posterior cervical soft tissues. Several studies over the past decade have demonstrated that cervical ligamentous injuries (predominantly the nuchal, atlanto-occipital and atlanto-axial ligaments) are frequent in abusive head trauma [35, 36]. Given that spinal injury mostly involves the ligamentous and soft tissues and only rarely bony injury, up to 90% of spinal injuries are missed on CT [30].
- The assessment of the cervical cord and its ability to visualise the remainder of the spinal cord and canal [51].
- To assess for the presence of concomitant retinal haemorrhages which may be associated with abusive head trauma but which may require a focused protocol [52–54].
- ‘Black bone’ [55] and newer volumetric sequences [56] are being increasingly investigated in the diagnosis of skull fracture with promising results when compared to CT but which still require validation.

Nevertheless, MRI has some limitations:

- There are technical and logistical limitations in infants and young children with motion artefacts related to the duration of MRI examination.
- Given the typical age for suspected physical abuse (less than 24 months), general anaesthetic or sedation will usually be required. Furthermore, access to MRI is not always possible in emergency situations or outside of normal working hours (institution dependent).
- The detection and interpretation of skull fractures and haemosiderin deposition can be challenging and are difficult to detect on conventional MRI sequences. The high sensitivity of SWI/GRE T2* sequences can generate difficulties in evaluating the precise extent of subdural or subarachnoid haemorrhage [57] alongside difficulties in determining the age of haemorrhage which remain complex for the (neuro)radiologist [58].

Following this discussion on the advantages and disadvantages of head CT and MRI in the imaging of (suspected) abusive head trauma, both modalities remain complimentary depending on the clinical context in which they are used. In children with an acute or subacute neurological presentation, CT remains the first-line imaging modality in the assessment of (suspected abusive) head injury followed by an early MRI. In asymptomatic children, e.g. siblings of an index child suspected of having been physically abused, or those having been presented with a suspected inflicted injury or isolated macrocrania, MRI can be the employed as the primary modality.

The optimal radiological screening of asymptomatic, but at risk contact children, particularly twins, needs further discussion with a view to standardisation [59]. At present, no clear consensus-based guideline exists on this subject. However, some of the authors of this paper recently led on a consensus-based study to fill this practice gap working with an international group of radiologists and child abuse paediatricians. The results of this study are currently in press and will be available soon. At present, MRI is recommended as the first-line imaging modality given its safety, higher yield for occult lesions, particularly in those most vulnerable under the age of 2 years, and it obviates exposure to ionising radiation. A standardised approach when investigating abusive head trauma in index patients and their at-risk siblings is advocated to protect both the institutions from the liabilities of missing or overcalling a diagnosis and those children at risk [59].
