# Correction to: Controversial aspects of imaging in child abuse: a second roundtable discussion from the ESPR child abuse taskforce

**DOI:** 10.1007/s00247-023-05646-1

**Published:** 2023-03-18

**Authors:** Michael Paddock, Arabinda K. Choudhary, Annmarie Jeanes, Kshitij Mankad, Inès Mannes, Maria Raissaki, Catherine Adamsbaum, Maria I. Argyropoulou, Rick R. van Rijn, Amaka C. Offiah

**Affiliations:** 1 Medical Imaging Department, Perth Children’s Hospital, Perth, WA Australia; 2 Division of Paediatrics, University of Western Australia, Perth, WA Australia; 3 Department of Oncology & Metabolism, University of Sheffield, Sheffield Children’s NHS Foundation Trust, Sheffield, UK; 4 Department of Diagnostic Radiology, University of Arkansas for Medical Sciences, Little Rock, AR USA; 5 Department of Paediatric Radiology, Leeds Children’s Hospital, Leeds Teaching Hospitals NHS Trust, Leeds, UK; 6 Department of Radiology, Great Ormond Street Hospital NHS Foundation Trust, London, UK; 7 Paediatric Radiology Department, AP-HP, Bicêtre Hospital, Le Kremlin-Bicêtre, Paris, France; 8 Radiology Department, Medical School, University Hospital of Heraklion, University of Crete, Crete, Greece; 9 Faculty of Medicine, Paris-Saclay University, Le Kremlin Bicêtre, Paris, France; 10 Department of Radiology, Medical School, University of Ioannina, Ioannina, Greece; 11 Department of Radiology and Nuclear Medicine, Emma Children’s Hospital, Amsterdam UMC, University of Amsterdam, Amsterdam, the Netherlands; 12 Department of Radiology, Sheffield Children’s NHS Foundation Trust, Sheffield, UK

**Correction to** **:** **Pediatric Radiology (2023)**

https://doi.org/10.1007/s00247-023-05618-5

The initial online version states that all authors participated in the roundtable discussion.

Correction:

Only the following authors participated in the roundtable discussion.

Chair: A.C.O.

Panel/speakers: A.M.J., K.M., I.M., M.R.

The original article has been corrected.

